# Aspects of coverage in medical DNA sequencing

**DOI:** 10.1186/1471-2105-9-239

**Published:** 2008-05-16

**Authors:** Michael C Wendl, Richard K Wilson

**Affiliations:** 1 Genome Sequencing Center and Department of Genetics, Washington University, St. Louis MO 63108, USA

## Abstract

**Background:**

DNA sequencing is now emerging as an important component in biomedical studies of diseases like cancer. Short-read, highly parallel sequencing instruments are expected to be used heavily for such projects, but many design specifications have yet to be conclusively established. Perhaps the most fundamental of these is the redundancy required to detect sequence variations, which bears directly upon genomic coverage and the consequent resolving power for discerning somatic mutations.

**Results:**

We address the medical sequencing coverage problem via an extension of the standard mathematical theory of haploid coverage. The expected diploid multi-fold coverage, as well as its generalization for aneuploidy are derived and these expressions can be readily evaluated for any project. The resulting theory is used as a scaling law to calibrate performance to that of standard BAC sequencing at 8× to 10× redundancy, i.e. for expected coverages that exceed 99% of the unique sequence. A differential strategy is formalized for tumor/normal studies wherein tumor samples are sequenced more deeply than normal ones. In particular, both tumor alleles should be detected at least twice, while both normal alleles are detected at least once. Our theory predicts these requirements can be met for tumor and normal redundancies of approximately 26× and 21×, respectively. We explain why these values do not differ by a factor of 2, as might intuitively be expected. Future technology developments should prompt even deeper sequencing of tumors, but the 21× value for normal samples is essentially a constant.

**Conclusion:**

Given the assumptions of standard coverage theory, our model gives pragmatic estimates for required redundancy. The differential strategy should be an efficient means of identifying potential somatic mutations for further study.

## Background

Applications of DNA sequencing to medically significant problems continue to grow [1-6]. In particular, recent technological trends suggest that the sequencing of entire cohorts of individual patient genomes will soon be economically feasible [7-13]. This contrasts dramatically with the enormous resources that were expended on deciphering just a single composite human reference genome only a few years ago [14]. Sequence-based characterization promises to play an expanding role in medicine because of its ability to identify potential disease-causing mutations [1]. It will be especially important in cancers, for example, for distinguishing between sequence variations in the germline versus somatic mutations that are relevant to tumor initiation or growth [1,2].

In principle, the process-engineering issues in both gene-based and whole-genome medical sequencing are identical to those for *de novo *genomic sequencing, that is, to "cover" a region of interest with shotgun read data. However, the definitions of what constitutes coverage are rather different. In traditional genomic sequencing, the target is a haploid genome and coverage of a base position *x *is defined as the event whereby one or more sequence reads span *x*. Such a process is binomial and, according to elementary probability theory, the expected fractional coverage is 1-exp(-*ρ*), where *ρ *= *NL/G*. Here, *L *and *G *are the read and haploid genome lengths, respectively, *N *is the number of reads sequenced, and *ρ *is the haploid redundancy. Although this result describes a number of traditional coverage configurations [15], it seems to be known to the sequencing community primarily via its application by Clarke and Carbon [16]. This expression is also sometimes attributed to Lander-Waterman Theory (LWT) [17], although LWT actually treats the issue of sequence gaps rather than coverage.

Medical sequencing projects focus on genetic variation and seek to identify both alleles at *x *for the diploid genome. In particular, diploid sequence is necessary for discerning heterozygous mutations. Consequently, coverage is thought of in a more general way. Here, we say that *x *is "covered" when each allele is spanned by at least *φ *reads, where *φ *≥ 1. Actual values of *φ *will depend upon study-specific considerations that weigh economic factors against such things as desired confidence levels for detection and confirmation, anticipated data quality, etc. Some results on multiple coverings appear in the mathematical literature [18,19], but these do not address the problem beyond the haploid level. Smith and Bernstein [20] conducted early numerical simulations for *φ *= 1 on a 20 kb fragment, but evidently did not extend the approach to genome-size targets. Levy et al. [12] and Wheeler et al. [13] also describe models for this problem, which we discuss further below.

An important issue for future medical sequencing projects can be posed as follows. Given a specific choice of *φ*, estimate the necessary redundancy such that either the probability of covering a given position, e.g. a SNP, has some desired value, or that the expectation for the number of captured positions has such a value. These propositions are actually identical (see Methods). However, additional study-specific issues also arise. For example, for tumor/germline pairs, one has to specify *ρ *for both types of samples. As we demonstrate below, the two values should not necessarily be the same.

Speculation regarding these issues has been around for some time. For example, Strausberg et al. [1] observed that *ρ *should exceed 10. In other words, redundancies for medical sequencing projects should surpass those values conventionally associated with haploid whole-genome shotgun projects, BAC projects, etc. This is largely intuitive, given the diploid nature of the problem, but not particularly informative. Pioneering diploid sequencing projects furnish some early anecdotal information. For example, Levy et al. [12] considered *ρ *= 20 to be adequate for germline sequencing of a healthy individual based upon simulation, certain heuristic filters, and the model alluded to above. They employed traditional Sanger sequencing [21] and reached only about 7.5×, so the degree to which this value generalizes to medical sequencing of cancer genomes using short-read "next-generation" platforms [8] is unclear. Likewise, Wheeler et al. [13] report only about 7.4× for another diploid project.

Here, we address medical sequencing coverage more formally by way of a straightforward mathematical extension to the standard covering process model. We consider this an idealization in the sense that it presumes all entities are independently and identically distributed (IID) and neglects any heuristic inputs. However, we also demonstrate the use of empirical data to calibrate response such that inferences can be drawn for medical sequencing projects. The resulting analysis points to what we believe will be an efficient means of discerning potential somatic mutations and enables estimation of the necessary parameters.

## Results

Given a location *x *defined in the context of *h *associated chromosomes, let *P*_*h*, *φ *_be the probability that *x *is covered at least *φ *times. The immediate focus of much of the research community is on diploid sequencing of homologous chromosomes (*h *= 2) related to the cancers, for which we report a mathematical theory of coverage. In anticipation of extending sequencing to aneuploid configurations, some of which are also relevant to cancer, we furnish the general result for *h *> 2, as well.

### Diploid Sequencing Theory

Given a diploid genome (*h *= 2), the probability of coverage is

(1) $$P_{2,\varphi }=\sum_{j=\varphi }^{N-\varphi }C_{N,j}\delta_{2}^{j}{\left(1-\delta_{2}\right)}^{N-j}\left[1-\sum_{k=0}^{\varphi -1}C_{N-j,k}\delta_{2}^{k}{\left(1-\delta_{2}\right)}^{N-j-k}\right],$$

where *δ*_2 _= *L*/(2*G*) is the diploid Bernoulli probability and *C*_*N*, *k *_are the binomial coefficients. Eq. 1 also gives the expected fraction of a set of locations that are covered (Methods). This equation relies on the standard IID assumption, but is exact in the sense that it accounts for the fact that the coverings of two corresponding alleles on homologous chromosomes are not strictly independent of one another (Methods). However, parameters in an actual project are such that alleles are *almost *independent. Moreover, asymptotic approximation can be applied (Methods), in which case

(2) $$P_{2,\varphi }\approx {\left(1-\sum_{k=0}^{\varphi -1}\frac{1}{k!}{\left(\frac{\rho }{2}\right)}^{k}e^{-\rho /2}\right)}^{2}$$

is a very good approximation of Eq. 1. Here, e is the Euler Number (≈ 2.71828) and *ρ *is again the conventional haploid redundancy. Note the basis in a Poisson distribution having a rate *ρ*/2. Eq. 2 is straightforward to evaluate for any project because *φ *is typically not very large. This stands in contrast to Eq. 1, which sports an enormous number of terms, as well as tendencies for numerical overflow and underflow of its various components. For convenience, we expand the first three expressions

(3) $$P_{2,1}\approx {\left(1-e^{-\rho /2}\right)}^{2}$$

(4) $$P_{2,2}\approx {\left(1-e^{-\rho /2}\left(1+\frac{\rho }{2}\right)\right)}^{2}$$

(5) $$P_{2,3}\approx {\left(1-e^{-\rho /2}\left(1+\frac{\rho }{2}+\frac{\rho^{2}}{8}\right)\right)}^{2}.$$

### Generalization to Aneuploidy

Under the assumption of independence, Eq. 2 for homologous chromosomes is readily generalized to an arbitrary number of chromosomes, *h*, specifically

(6) $$P_{h,\varphi }\approx {\left(1-\sum_{k=0}^{\varphi -1}\frac{1}{k!}{\left(\frac{\rho }{h}\right)}^{k}e^{-\rho /h}\right)}^{h}.$$

Note the Poisson basis having a rate *ρ*/*h*, for example *ρ*/3 for chromosomal trisomies. Like Eq. 2, this expression is readily evaluated and straightforward to expand for given values of *φ*.

## Discussion

A number of medical sequencing coverage issues are currently being debated. New questions have arisen not only because diploid medical sequencing is itself a fairly recent undertaking, but also because of the expectation that novel sequencing platforms will be heavily employed in such projects. Read lengths are substantially shorter than traditional Sanger data [21] and investigators are eager to determine how this affects coverage. We focus our discussion here primarily on the diploid problem, although some projections for aneuploid configurations are given, as well.

### Coverage Assessment

**Figure 1:**
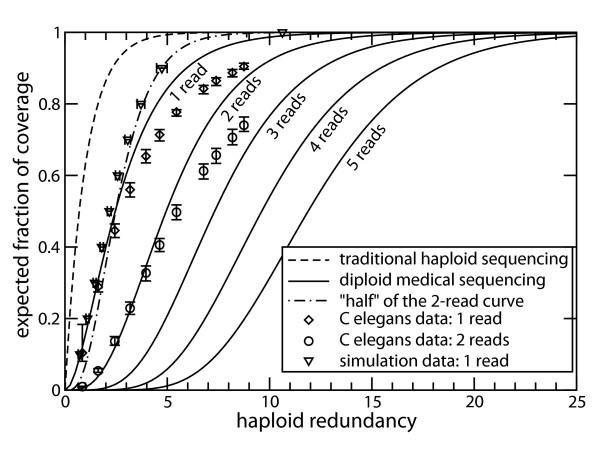
**Traditional haploid coverage model [15, 16] versus diploid medical sequencing coverage results for minimum number of covering reads *φ *∈ {1, 2, 3, 4, 5}.** The figure also shows an additional curve that replots the diploid *φ *= 2 curve, except where abscissa values are scaled by one-half. This aspect is relevant to the discussion of why the redundancies for *φ *= 1 and *φ *= 2 do not differ by a factor of two. Coverage progressions for *φ *∈ {1, 2} are also shown for the recent Illumina resequencing of *C. elegans *by Hillier et al. [28]. These points represent average coverages over all chromosome pairs, while their error bars show the observed minima and maxima. Simulation data for *φ *= 1 on a 20 kb fragment using 250 bp reads [20] are also shown. Points and error bars represent the averages and extrema, respectively, of 250 simulations.

Fig. 1 shows the traditional *de novo *haploid coverage model [15,16] versus diploid medical sequencing coverage theory for minimum read coverings of *φ *∈ {1, 2, 3, 4, 5} for both alleles. The diploid curves were generated by Eq. 2 for a 3.3 billion base-pair genome and 31 base-pair read lengths. Errors associated with not using Eq. 1 for these particular parameters are significantly less than 1% (data not shown). As one would intuitively expect, the required redundancies for a given coverage fraction increase with *φ *and are noticeably higher than established values for haploid genome sequencing. Although these cases are within the realm of feasibility for the newest-generation sequencing platforms [11], economic factors would probably still preclude the higher values of *φ *at the present time. Conversely, these depths are much lower than values that have been discussed elsewhere. For example, Warren et al. [22] report *ρ *up to 100 and 400 for bacterial and viral genomes, respectively, using 25 bp fragments to simulate data from an Illumina instrument.

Two other notable trends are visible in Fig. 1. First, increasingly large redundancies are required just to obtain non-trivial values of *P*_2, *φ*_. For example, the curve for *φ *= 1 already exceeds 0.01 at *ρ *= 0.25, whereas this mark is not met until *ρ *= 5 at *φ *= 5. Indeed, one may not see even the "beginnings" of coverage until comparatively high redundancy has been reached, depending on the selected *φ*. Also, the amount by which each curve is drawn-out over the abscissa increases with *φ*, signifying a decelerating coverage rate. This is especially clear for what appear to be the linear segments of each curve; their slopes progressively decrease. Again comparing the extremes, the difference between 0.1 and 0.9 on the ordinate is a little over 5 units of redundancy for *φ *= 1 but almost 11 units for *φ *= 5. This phenomenon bears on point we make below.

Both of these trends arise strictly as mathematical consequences and can perhaps best be understood by referring to Eqs. 3 through 5. The exponential (Euler Number) term represents the tendency for the coverage rate to decay. For each successive value of *φ *this term is bolstered by additional factors, which themselves grow progressively faster with *ρ*, whereby the overall effect is realized.

### Calibration and the Stopping Problem

One of the primary issues facing the investigator is the so-called stopping problem. That is, at what *ρ *should random processing be halted? This question is, of course, context dependent. Yet, it can be answered, at least approximately, by using the analysis given here. For example, suppose the goal is to design a medical sequencing project such that the expected coverage progress corresponds roughly to standard BAC sequencing. This is a calibration-based way of framing the question and exploits the community's collective empirical experience gained from having sequenced hundreds of thousands of such clones. In particular, 6 ≤ *ρ *≤ 10 has been found to be a reasonable balance between cost and coverage, although values nearer to 10 are more typically chosen [14]. In this capacity, Eqs. 1 and 2 effectively function as scaling laws.

**Figure 2:**
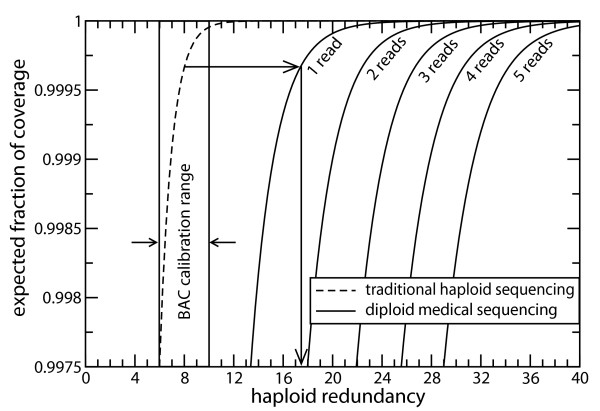
**Haploid and diploid results for expected coverage values of at least 0.9975.** This is a greatly – magnified view of the top quarter – percent of the ordinate range in Fig. 1. Vertical lines demarcate the typical BAC calibration neighborhood of 6 ≤ *ρ *≤ 10. The scaling process is demonstrated graphically for diploid sequencing (*φ *= 1) based on haploid sequencing at *ρ *= 8.

Scaling can conveniently be demonstrated graphically, for example by picking a point on the haploid curve for a desired redundancy, drawing a horizontal line through this point, and reading the redundancy at the intersection of the chosen diploid curve and the horizontal. The asymptotic nature of the curves depicted in Fig. 1 obscures this process, but it can readily be accomplished using a magnified plot. Fig. 2 shows the example of extrapolating haploid sequencing coverage at *ρ *= 8 to diploid sequencing (*φ *= 1), the result being about 17.5× for the diploid project. Table 1 furnishes an expanded set of values for *φ *∈ {1, 2, 3} calibrated against haploid sequencing for *ρ *∈ {6, 8, 10}. Again, the increase of redundancy with the minimum number of reads required to attain coverage is quite clear. Notice that each of the three rows in the table corresponds to covering more than 99% of the unique sequence. In other words, the covering probabilities change very little over fairly significant increases in depth. Consequently, BAC depth provides much better resolution than BAC coverage for scaling the diploid problem. This observation is also obvious from Fig. 2.

**Table 1:** Calibration of medical sequencing according to traditional haploid expectation

Traditional [16]		Corresponding Medical Sequencing *ρ*		
	
Redundancy	*P*-value	1-read min.	2-read min.	3-read min.
6	0.99752	13.5	18	22
8	0.99967	17.5	22.5	26.5
10	0.99996	21.5	26.5	31.5

### Comparison to Haploid-Based Distribution Models

Diploid coverage, as discussed above, is a primary consideration for medical sequencing. Yet, it is also useful for comparison to examine such projects in their haploid context. The stopping problem has been extensively studied from a number of analytical perspectives for traditional *de novo *genomic sequencing projects, for example using the probability of complete coverage, *P*_*C *_[23] and the intersection probability, *P*_⋂ _[24]. While the meaning of the former is probably clear, the latter characterizes how effective additional redundancy will be for improving coverage in light of increasingly-important stochastic effects. Of these two metrics, *P*_*C *_is the more conservative and could be thought of as setting an upper bound in the context of traditional haploid sequencing. Before proceeding, let us digress briefly to further explain *P*_⋂_.

**Figure 3:**
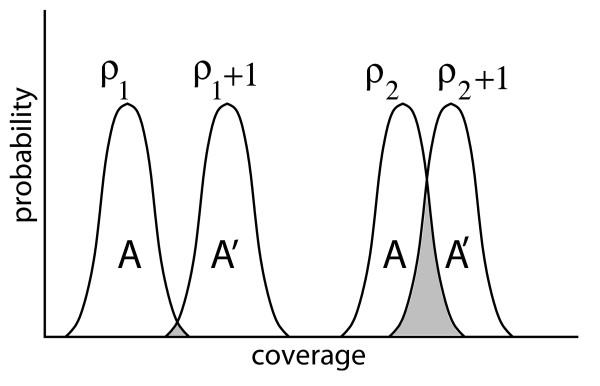
**Diagrammatic synopsis of the intersection probability.** Paired coverage distributions, plotted at differences of one unit of redundancy, begin to coalesce as a project evolves. The intersection probability is the area of the overlap (shaded).

Consider two hypothetical sequencing projects, *A *and *A*', that are identical in every respect, except that *A' *is always ahead by one whole unit of redundancy (Fig. 3). Now, examine these projects at two particular instances, specifically for project *A *at both *ρ*_1 _and *ρ*_2 _(with *A' *at *ρ*_1 _+ 1 and *ρ*_2 _+ 1, respectively), where *ρ*_1 _<*ρ*_2_. At *ρ*_1_, there may be little overlap in the densities between the two projects. Accordingly, the probability is extremely high, perhaps close to 1, that *A' *will be more highly covered than *A*. The system behaves as if there is a deterministic increase in coverage for *A *→ *A' *for a unit increase of redundancy. At higher redundancies, say *ρ*_2_, mathematical analysis indicates that the intersection of the *A *and *A' *densities will grow [24]. Consequently, differences in actual coverage between *A *and *A' *become progressively more a function of chance than of differences in the redundancies themselves. The tail value of the intersection, *P*_⋂_, can be taken as an indicator on the diminishing returns of the process.

We can once again take a calibration approach to this problem. That is, we calculate *P*_*C *_and *P*_⋂ _for a typical BAC sequencing project (150 kb clone length and 600 bp read length) at *ρ *∈ {6, 8, 10}. We then match these values to their counterparts in the distributions for medical sequencing, which then provides the corresponding "scaled" redundancy. Table 2 shows these results. The values compliment those in Table 1 in the sense that they suggest redundancies far above the conventional full shotgun standard of *ρ *= 10.

**Table 2:** Calibration of medical sequencing according to haploid distribution models

Traditional BAC Sequencing Project			Medical Sequencing *ρ *based on	
	
Redundancy	*P*_*C*_	*P*_⋂_	Complete Covg.	Intersection
6	0.02843	0.74122	20.2	18.8
8	0.51842	0.95054	22	20.9
10	0.89840	0.99301	23.9	23

### Comparison to Empirical, Semi-Empirical, and Simulation Results

Several labs are now involved in diploid sequencing projects, which should furnish useful examples of coverage progressions that can be monitored empirically. Although the few stopping redundancies reported in the literature appear to conflict with one another, these can be more properly interpreted according the minimum number of times each allele is observed, *φ*. For example, we mentioned above that Levy et al. [12] considered *ρ *= 20 for Sanger-based [21] germline sequencing of a healthy individual. This figure approaches the 10× standard for haploid sequencing [25] at the level of *φ *= 1 (Table 1). Interestingly, the idealized version of their coverage calculation proves to be a special case of our model precisely for *φ *= 1 (see Appendix). Conversely, Mardis [8] quotes a redundancy up to 30×, which corresponds to values of *φ *between 2 and 3 when calibrated to haploid 10×.

Richard Durbin and Aylwyn Scally have also analyzed the diploid medical sequencing coverage problem using a different approach from what is described here (Durbin and Scally, personal communication). Specifically, they employed an "extra-variation" Poisson distribution [26,27] having a free-parameter to control variance. Values for this parameter can be chosen to *a posteriori *tune the theoretical fit with empirical data. In particular, such tuning allows one to implicitly consider, at least approximately, factors such as bias and sequencing errors. (In our method, calibration incorporates the empirics of BAC sequencing, essentially serving the same purpose.) Using their semi-empirical approach, Durbin and Scally concluded that redundancies closer to 30× will be required, which again agrees well with results shown in Tables 1 and 2.

A number of labs, including our own, are now adopting "next generation" short-read sequencing technology [8] and have started to generate human medical sequencing data related to various cancers. However, there is still a dearth of published results from which actual coverage progressions can be derived. For the purposes of comparison, we refer instead to the recently completed pilot resequencing project for *C. elegans*. Hillier et al. [28] resequenced strain N2 Bristol using the Illumina Genome Analyzer in order to characterize the accuracy and utility of short-read, massively parallel data. We have projected their *C. elegans *coverage results for *φ *∈ {1, 2} onto Fig. 1. Agreement is very good up to about 60% coverage, after which the rate of empirical coverage falls below expectation. This behavior seems to typify theoretical-empirical differences. For example, Wendl and Barbazuk [29] noted precisely this trend for sequencing filtered genomes.

The physical explanation of this phenomenon is straightforward. Specifically, biases are not manifested early in a project because there is not enough information to distinguish unbiased coverage configurations from biased ones. (Think of an extreme case, for example placing a single read of high GC content onto a genome of high AT content. Despite the obvious non-IID nature of this scenario, the predicted coverage will still be *identical *to the actual coverage.) Given a model based on the IID assumption, as ours is, empirical and theoretical results should start to diverge as sufficient information gathers to expose latent biases. In this case, Hillier et al. [28] note a definite AT bias using the Illumina platform, i.e. remarkably lower coverage in regions of high AT content, which we presume accounts for much of the difference shown in Fig. 1. The proclivities of other methods and platforms are evidently different [30]. Consequently, Eq. 2 should also be useful as a yardstick for comparison among these approaches for specific applications.

Finally, Fig. 1 also shows simulation data reported by Smith and Bernstein [20] for *φ *= 1 on a 20 kb circularized fragment using 250 bp reads. Agreement is once again good up to about 60% coverage, after which the sequencing process seems to grow more efficient for the fragment. This observation is not surprising, given two important aspects of this study. First, the circularized configuration is not subject to the so-called "edge effect", which can dramatically affect coverage rates [29,31]. Second, distribution theories show that configurations having larger *L*/*G *ratios do indeed cover more readily than those having smaller values [24]. We presume these two factors account for most of the difference, especially given that *L*/*G *= 0.0125 for the simulation is more than a million times larger than values associated with short-read medical sequencing projects. For example, 31 bp reads on a 3.3 billion bp genome yields *L*/*G *≈ 1 × 10^-8^.

### A Differential Sequencing Strategy

We expect that many future studies will be based on sequencing DNA derived from matched tumor/normal samples (for example, the latter being obtained from uninvolved skin or blood) from the same patient [3,4]. Here, the whole genome of each sample in a pair is sequenced and mutations are found by comparison to the human reference. Let us call the sets of mutations for a tumor and a normal sample *S*_*T *_and *S*_*N*_, respectively, where we generally expect *S*_*N *_⊆ *S*_*T*_. Most of the germline variation in *S*_*N *_will be polymorphisms not related to pathogenesis [1], whereas *S*_*T *_will contain a potentially more relevant collection of somatic mutations. In principle, germline sequence variations can be removed from further consideration by taking the difference *S*_*T *_- *S*_*N *_[6]. Such filtering will appreciably focus subsequent work, since the overwhelming majority of sequence variants should be polymorphisms found in normal tissue [5]. How does one efficiently accomplish this from a process-engineering standpoint?

We propose a refinement of simple subtraction [6] in the form of a straightforward differential sequencing strategy. In principle, false-negative errors are controlled by sequencing at least to diploid coverage at the level of *φ *= 1. However, tumor samples should actually be sequenced as heavily as economically possible in order to minimize false-positive hits for both germline and somatic mutations. These types of mistakes arise, for example, by misinterpreting a random sequencing error as a true mutation. Given current state-of-the-art capabilities, we will assume this condition translates to diploid coverage at the level of *φ *= 2, but emphasize that future instruments will undoubtedly permit higher *φ*.

Conversely, a germline mutation in a normal sample only has to be detected once in order to be eliminated from *S*_*T*_. We are also not as concerned about false-positives here because their appearance in *S*_*N *_does not affect the subtraction *S*_*T *_- *S*_*N*_. It is possible that an error could lead to a spurious entry in *S*_*N *_that precisely matches a true somatic mutation in *S*_*T *_by pure chance. The somatic mutation would then be erroneously eliminated from further investigation. However, such events seem unlikely, given the low anticipated number of bona fide somatic mutations. These observations collectively imply that normal samples may only need to be sequenced to diploid coverage at the level of *φ *= 1.

The 10× standard for BAC sequencing is well-established [25] and provides a reasonably conservative basis to translate the above design into actual redundancies for medical sequencing (Table 1). We suggest then that sequencing of tumor samples should not be pursued to less than about 26.5× redundancy, given the 2-read minimum coverage condition. Furthermore, paired normal samples need only to be sequenced to about 21.5× redundancy for the *φ *= 1 coverage condition.

### Expository Comment on Recommended Redundancies

The observation that the required redundancy for the *φ *= 1 coverage level is not simply half that of the *φ *= 2 level may initially seem counter-intuitive. We remarked above that curves for increasing values of *φ *tend to have progressively smaller slopes. Consequently, there is not, in general, an integer-valued relationship between corresponding points on any two particular curves. In other words, curves are not simply shifted along *ρ*. Returning to Fig. 1, we show an example that replots the *φ *= 2 curve, except where the abscissa-value of each of its points has been divided by two. It is clear that the result does not coincide with the *φ *= 1 curve, as intuition may have suggested. The curves only intersect at a single point, here at an expected coverage slightly more than 0.5, which is well below the > 0.99 calibration points we chose above. In other words, redundancy for *φ *= 1 would only have been half that for *φ *= 2 had we chosen to cover 50% instead of > 0.99 of SNPs. This trend holds generally. That is, we do not expect an integer relationship between the required redundancies for two unequal, but otherwise arbitrary values of *φ*.

### Coverage Projections for Aneuploid Configurations

**Figure 4:**
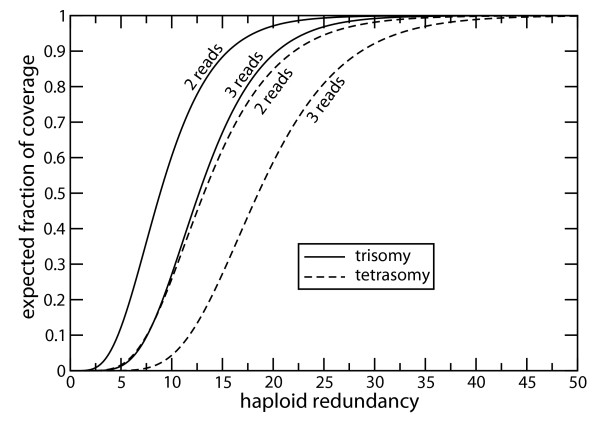
Expected coverage for aneuploid chromosome configurations for minimum number of covering reads *φ *∈ {2, 3}.

Aneuploidy can be manifested in a number of ways: as an autosomal [32] or sex chromosome [33] aberration, and in conjunction with cancer [34]. We anticipate the eventual application of DNA sequencing to aneuploid chromosome configurations and offer some early projections based upon Eq. 6. Fig. 4 shows expected coverage for trisomy and tetrasomy for *φ *= 2 and *φ *= 3. Required depths are clearly much higher than for diploid sequencing. For example, we find redundancies of *ρ *= 42 and *ρ *= 57 for trisomy and tetrasomy, respectively, when scaling to 10× BAC sequencing at the level of *φ *= 2. Recall that *φ *= 2 is presumed to be feasible for diploid whole-genome sequencing using current hardware. These redundancies are clearly out of reach at the moment for a whole-genome project, but may be feasible for chromosome-specific projects. In other words, the appreciably higher cost of sequencing aneuploid chromosomes may justify the effort of separating them into their own self-contained projects.

### Modeling Limitations

As with the classical theories of sequencing [16,17], the main assumption here is that reads are independently and identically distributed (IID). In other words, this analysis does not formally consider biological or instrument-specific biases, software biases and sequencing errors, for example in base-calling, assembly problems, or any other heuristic inputs. The idiosyncrasies of each of these factors are difficult to characterize analytically, although the calibration step does allow some implicit accounting, as noted above. Appreciable differences in levels and types of bias have been noted, for instance in Sanger-style sequencing versus pyrosequencing [30], so any results should be interpreted with these qualifications in mind.

In general, the assumption of allele independence should be valid for most medical sequencing projects since *φ *will be small and *L*/*G *→ 0 and *N *≫ 1 (see Methods). For example, maximum error for *φ *∈ {1, 2} is on the order of 10^-5 ^percent for diploid sequencing using 650 bp read lengths. The theory further assumes that sequence reads have no preference for either chromosome of a homologous pair and neglects any tendency for reads to align to multiple positions. The latter has been found to occur with some frequency if reads are short enough [35]. Read-pairing certainly curtails this phenomenon, but the pairing process itself has negligible effect on coverage unless the target is very small [31]. Such is not the case in whole-genome medical sequencing, so the net effect of pairing is simply that the amount of uniquely-alignable sequence one gets to count toward *ρ *increases commensurately. In plain terms, fewer data will be discarded.

Finally, our analysis does not account for what might be called the "uneven coverage" problem of alleles. Mutation detection programs may decline calling out a SNP if one allele is covered much more heavily than the other [36]. Because this phenomenon is both software-specific and sequence-specific, it is beyond our scope. Departure from any of these idealizations will tend to reduce coverage, implying that our analysis is best viewed in the context of upper bounds of performance. In other words, required redundancies for specific projects may still exceed what we have advocated here, as the *C. elegans *data in Fig. 1 illustrate.

A subtle mathematical point is also worth mentioning. Eqs. 1 through 5 represent the probability of covering a specific allele pair, or alternatively, the expected fraction of pairs covered. These expressions do not provide the underlying distribution of the number of covered pairs, which is a more formidable mathematical problem. In other words, this is a model only of coverage expectation, exactly analogous to what classical theories [16,17] are for traditional *de novo *haploid sequencing. Consequently, the results themselves are not strong functions of *L*/*G*. In fact, for most applications the results will be completely independent of this ratio and will instead follow a set of "universal" curves, the first 5 of which are shown in Figs. 1 and 2. This point is underscored by Eq. 2, which is a function strictly of *ρ*. (The observation holds more generally for aneuploidy as described by Eq. 6, as well.) This phenomenon contrasts with distribution-based models, such as *P*_*C *_and *P*_⋂ _discussed above, which are indeed sensitive to *L*/*G*. The basis of this effect is discussed in ref. [24].

A corollary to this observation is that adjusting the fundamental parameters within their biologically-relevant limits will have no effect on the results we have discussed. For example, the haploid genome size *G *could be adjusted to reflect only that part of the sequence to which read data can be uniquely aligned [35]. Yet, the underlying assumptions leading to Eqs. 2 and 6 will still be satisfied in this circumstance, mainly *L*/*G *→ 0 and *N *≫ 1 (Methods). The same holds for varying *L *in order to represent different kinds of sequencing platforms, e.g. pyrosequencing or Sanger instruments. In summary, the contributions of the three independent variables *L*, *G*, and *N *collapse into the single dimensionless variable *ρ*, which governs the process exclusively. Formal theory [37] predicts such systematic reductions of variables whenever a unified dimensionless parameter lurks in a problem.

## Conclusion

The differential sequencing strategy should be useful for efficiently identifying lists of somatic mutations for validation and further study. Our analytical model of coverage, coupled with a calibration approach for selecting parameters, allows pragmatic estimates to be made for such projects. However, because the theory does not strictly consider various biasing factors, actual projects would benefit from periodically aligning (assembling) shotgun data to empirically track overall coverage, as well as local coverage in coding regions, UTRs, promoters, and conserved regions. SNP arrays could also be done for each sample, with attempts made to find and correlate data to sequence calls for further coverage tracking. Plotting these various data on a single figure, as we did for the *C. elegans *data in Fig. 1, should be informative. Finally, the basic model could be further extended in the future as more data accrue from different methods, projects, software processing pipelines, etc. For example, "extra-variation" methods [26,27] could be used for *a posteriori *data fitting, the results of which should help to better quantify non-IID factors.

## Methods

Proofs of Eqs. 1, 2, and 6 are reported here. Eqs. 3 through 5 follow trivially from Eq. 2. We also describe the analysis of *C. elegans *resequencing data from Hillier et al. [28].

### Preliminaries

Let *B*_*i*, *j *_be the event where an allele at position *x *on chromosome *i *is "covered", i.e. spanned by at least *φ *out of any collection of *j *reads, where *j *≥ *φ*. Given *N *total reads, our definition of diploid medical sequencing coverage for position *x *is then *B*_1, *N *_⋂ *B*_2, *N *_and its probability is *P*_2, φ_(*B*_1, *N *_⋂ *B*_2, *N*_). If *β*_*i*, *j*, *k *_is the event whereby the allele on chromosome *i *is spanned by exactly *k *of *j *reads, then *B*_*i*, *j *_≡ *β*_*i*, *j*, *φ *_⋃ *β*_*i*, *j*, *φ*+1 _⋃ ⋯ ⋃ *β*_*i*, *j*, *j*_. Considering two homologous chromosomes, *i *∈ {1, 2}, the probability that a single given read spans *x *on a specific chromosome is *δ*_2 _= *L*/(2*G*), where *L *and *G *are read length and haploid genome length, respectively. Since the process is binomial (covering or not covering), we immediately have $P(\beta_{i,j,k})=C_{j,k}\delta_{2}^{k}{\left(1-\delta_{2}\right)}^{j-k}$, where *C*_*j*, *k *_are the binomial coefficients.

### Proof of Eq. 1

The coverings of two homologous alleles are not independent of one another. For instance, if one allele is already covered by *j *reads, there are only *N *- *j *remaining reads that have a chance to cover the other allele. Consequently,

$$P_{2,\varphi }(B_{1,N}\cap B_{2,N})=P(B_{1,N})\cdot P(B_{2,N}|B_{1,N})=\sum_{j=\varphi }^{N}P(\beta_{1,N,j})\cdot P(B_{2,N-j}|\beta_{1,N,j}),$$

where

$$P(B_{2,N-j}|\beta_{1,N,j})=\sum_{k=\varphi }^{N}P(\beta_{2,N-j,k})=1-\sum_{k=0}^{\varphi -1}P(\beta_{2,N-j,k}).$$

Eq. 1 follows from the observation that *P*(*β*_2, *N*-*j*, *k*_) = 0 for *k *> *N *- *j*.

### Proof of Eq. 2

If we neglect the dependence of alleles, then *P*_2, *φ*_(*B*_1, *N *_⋂ *B*_2, *N*_) = *P *(*B*_1, *N*_)·*P *(*B*_2, *N*_). Without loss of generality, this probability is identical to *P*^2^(*B*_1, *N*_), from which

$$P_{2,\varphi }={\left(1-\sum_{k=0}^{\varphi -1}C_{N,k}\delta_{2}^{k}{\left(1-\delta_{2}\right)}^{N-k}\right)}^{2}.$$

Parameters for medical sequencing projects are such that *L*/*G *→ 0 and *N *≫ 1. These relations hold for both "short read" platforms and instruments that provide "full-length" Sanger read data. Moreover, *φ *≪ *N*, which implies *k *≪ *N*. Consequently, the binomial coefficients *C*_*N*, *k *_are well-approximated by *N*^*k*^/*k*!. These conditions also imply (1 - δ_2_)^*N *^~ exp(-*Nδ*_2_), i.e. that asymptotic approximation can be used for the power term. Eq. 2 follows directly.

### Proof of Eq. 6

The case of aneuploidy under the assumption of allele independence is a straightforward extension of the proof for Eq. 2. For *h *homologous chromosomes, *P*_*h*, *φ *_(*B*_1, *N *_⋂ *B*_2, *N *_⋂ ⋯ ⋂ *B*_*h*, *N*_) = *P*(*B*_1, *N*_)*P *(*B*_2, *N*_) ⋯ *P *(*B*_*h*, *N*_) = *P*^*h*^(*B*_1, *N*_). Given that all *h *chromosomes are equally likely to be sampled, *δ*_*h *_= *L*/(*hG*), which is the appropriate Bernoulli probability for *P*(*B*_1, *N*_). Eq. 6 follows from the same approximation arguments made for Eq. 2.

### Eqs. 1, 2, and 6 in the Context of Expectation

We can take the coverage status of a specific allele pair as a Bernoulli trial, whereby elementary probability theory shows that the expected number of pairs covered is their total number multiplied by *P*_2, *φ*_(*B*_1, *N *_⋂ *B*_2, *N*_). Consequently, *P*_2, *φ*_(*B*_1, *N *_⋂ *B*_2, *N*_) in Eq. 1 and its approximation in Eq. 2 also represent the expected *fraction *of covered pairs. The same argument holds for Eq. 6.

### Analysis of *C. elegans *resequencing data

Hillier et al. [28] used the Illumina Genome Analyzer to resequence the *C. elegans *N2 Bristol genome. Release ws188 of the genomic sequence [38] was downloaded from  and randomly chosen subsets of the resequence data were aligned against the reference at regular intervals for each chromosome using the maq aligner (). Data that could not be uniquely placed on the reference were discarded. Coverage was calculated for each alignment as the number of corresponding base positions spanned by at least one read on homologous chromosomes (*φ *= 1) and by at least two reads on homologous chromosomes (*φ *= 2).

## Appendix: Idealized Theory of Levy et al

Levy et al. [12] sketch a rudimentary diploid theory, though they do not furnish any corresponding mathematical description. Here, we reconstruct an idealized version of their model, i.e. the form which assumes all entities are IID and which omits any heuristic inputs. A careful reading of "Modeling False-Negative Rate of Heterozygous Variants" in ref. [12] reveals the following salient features. Chromosomes are equally likely to be sampled and loci are taken as independent of one another. (Our theory relies on these same two assumptions.) Levy et al. also assume the number of reads *ν *spanning a position of interest *x *is Poisson-distributed with a rate *ρ *and that the probability of observing both alleles is a binomial function of *ν*. Incidentally, Richard Durbin and Aylwyn Scally discuss a similar model in their analysis (Durbin and Scally, personal communication), as do Wheeler et al. [13].

Let the random variables **B **and **N **be the events where both alleles at *x *are observed (covered) and where *ν *reads span *x*, respectively. We immediately have

(7) $$P(N=\nu )=\frac{e^{-\rho }\rho^{\nu }}{\nu !}$$

from the Poisson assumption. Given *ν *reads spanning *x*, the probability of observing both alleles is simply the complement of the probability of any configuration in which one of the alleles is *not *represented among the *ν *reads. If we label the alleles **I **and **II**, then without loss of generality, the binomial model for the number of observations, *j*, of allele **I **is

(8) $$P_{I}(j)=C_{\nu ,j}{\left(\frac{1}{2}\right)}^{j}{\left(1-\frac{1}{2}\right)}^{\nu -j}=C_{\nu ,j}{\left(\frac{1}{2}\right)}^{\nu }.$$

There are two configurations in which only one of the alleles is observed: *j *= 0 (all reads hit allele **II**) and *j *= *ν *(all reads hit allele **I**). Consequently, the probability of observing both alleles in *ν *reads is *P*(**B**|**N **= *ν*) = 1 - *P*_**I**_(0) - *P*_**I**_(*ν*). Using Eq. 8, a little algebra shows

(9) $$P(B|N=\nu )=1-{\left(\frac{1}{2}\right)}^{\nu -1},$$

which is defined for *ν *≥ 1. Note that the probability exceeds zero only for *ν *≥ 2, as we would expect. That is, at least 2 reads must span *x *before it is possible to observe both alleles. The Theorem of Total Probability now furnishes the desired result, *P*(**B**), from Eqs. 7 and 9, as follows.

(10) $$\begin{array}{c} P(B)=\sum_{\nu }P(B|N=\nu )P(N=\nu )\\ =\sum_{\nu =2}^{\infty }\left[1-{\left(\frac{1}{2}\right)}^{\nu -1}\right]\frac{e^{-\rho }\rho^{\nu }}{\nu !}. \end{array}$$

This expression represents the ideal probability of covering a diploid location as a function of the haploid sequence redundancy of the project.

Eq. 10 is actually just a special case of our model in Eq. 2 for *φ *= 1, as the following exercise demonstrates.

(11) $$P(B)=\sum_{\nu =0}^{\infty }\left[1-{\left(\frac{1}{2}\right)}^{\nu -1}\right]\frac{e^{-\rho }\rho^{\nu }}{\nu !}-\sum_{\nu =0}^{1}\left[1-{\left(\frac{1}{2}\right)}^{\nu -1}\right]\frac{e^{-\rho }\rho^{\nu }}{\nu !}$$

(12) $$=\sum_{\nu =0}^{\infty }\frac{e^{-\rho }\rho^{\nu }}{\nu !}-\sum_{\nu =0}^{\infty }\frac{e^{-\rho }\rho^{\nu }}{\nu !2^{\nu -1}}-(1-2)e^{-\rho }$$

(13) $$=1-2e^{-\rho /2}\sum_{\nu =0}^{\infty }\frac{e^{-\rho /2}{\left(\rho /2\right)}^{\nu }}{\nu !}+e^{-\rho }$$

(14) = 1 - 2*e*^-*ρ*/2 ^+ *e*^-*ρ*^

(15) $$P(B)={\left(1-e^{-\rho /2}\right)}^{2},$$

which is nothing more than *P*_2,1 _in Eq. 3. It can be shown along very similar lines that the Poisson/Binomial model outlined by Wheeler et al. [13] is also a special case of Eq. 2 for *φ *= 2, i.e. it leads to *P*_2,2 _(Eq. 4).

## Authors' contributions

Both authors framed the original problem. MCW conceived and constructed the mathematical theory and wrote the paper. Both authors read and approved the final manuscript.
